# Assessing physical fitness adaptations in collegiate male soccer players through training load parameters: a two-arm randomized study on combined small-sided games and running-based high-intensity interval training

**DOI:** 10.3389/fphys.2024.1466386

**Published:** 2024-09-11

**Authors:** YanXiu Quan, YongXing Zhao, Rabiu Muazu Musa, Ryland Morgans, Rui Miguel Silva, Chin-Hwai Hung, Yung-Sheng Chen

**Affiliations:** ^1^ College of Physical Education, China West Normal University, Nanchong, Sichuan, China; ^2^ College of Physical Education, Chizhou University, Chizhou, Anhui, China; ^3^ Centre for Fundamental and Continuing Education, Universiti Malaysia Terengganu, Kuala Nerus, Terengganu, Malaysia; ^4^ Faculty of Applied Social Sciences, Universiti Sultan Zainal Abidin, Kuala Terengganu, Malaysia; ^5^ Football Performance Hub, University of Central Lancashire, Preston, United Kingdom; ^6^ Escola Superior de Desporto e Lazer, Instituto Politécnico de Viana do Castelo, Rua Escola Industrial e Comercial de Nun’Álvares, Viana doCastelo, Portugal; ^7^ Research Center in Sports Performance, Recreation, Innovation and Technology (SPRINT), Melgaço, Portugal; ^8^ Department of Physical Education, Fu Jen Catholic University, New Taipei, Taiwan; ^9^ Department of Exercise and Health Sciences, University of Taipei, Taipei, Taiwan; ^10^ Exercise and Health Promotion Association, New Taipei, Taiwan; ^11^ Tanyu Research Laboratory, Taipei, Taiwan

**Keywords:** football, training load, drill-based games, physical fitness, athletic performance

## Abstract

**Objective:**

To evaluate the effects of a 4-week intervention combining small-sided games (SSGs) and high-intensity interval training (HIIT) on physical fitness in collegiate male soccer players.

**Methods:**

Twenty-one soccer players were randomly assigned to either the HIIT + SSGs group (n = 11) or a control group (n = 10). Physical fitness was assessed at baseline and 1-week post-intervention, including countermovement jump (CMJ), change of direction (COD) test, sprint test, repeated sprint ability (RSA) test, and 30–15 Intermittent Fitness Test (30-15IFT). The intervention comprised eight sessions over 4 weeks: four SSGs and four HIIT.

**Results:**

The intervention group showed small to moderate improvements: mean RSA improved by 4.5% (*p* = 0.07), CMJ increased by 3.2% (*p* = 0.12), and 30–15IFT scores enhanced by 6.8% (*p* = 0.09). Key predictors of group membership included heart rate load per minute (OR 1.602) and various GPS variables.

**Conclusion:**

The 4-week intervention combining SSGs with HIIT did not produce statistically significant improvements in most physical fitness variables compared to the control group. Although there were positive trends in variables such as RSA and 30-15IFT, these changes were modest and not statistically significant. The results suggest that while the combined SSGs and HIIT approach shows potential, its impact on physical fitness over a 4-week period is limited, with some variables, like CMJ, even showing decreases.

## Introduction

Small-sided games (SSGs) are drill-based training exercises that aim to replicate the dynamics of a real match by implementing task constraints to emphasize specific technical, tactical, and physical/physiological objectives (Davids et al., 2013; Clemente et al., 2021a). Extensive research has established SSGs as a popular and effective form of exercise for soccer players (Moran et al., 2019), providing players with a high level of physiological stimulus while engaging in dynamic drill-based activities. This approach allows coaches to integrate various stimuli and target specific tactical and technical aspects of the game (Clemente et al., 2020).

SSGs are typically categorized into three formats: small (1v1 to 4v4), medium (5v5 to 8v8), and large (9v9 to 11v11) (Owen et al., 2014). The smaller SSGs formats impose a demanding physiological stimulus, often exceeding 85% of the maximal heart rate, and place considerable mechanical work, acceleration, and deceleration demands on the players (Lacome et al., 2017). Due to the metabolic and neuromuscular benefits presented by these SSGs, this soccer-specific training mode is frequently utilized to promote maximal aerobic capacity and enhance endurance (Lacome et al., 2017). Notably, SSGs offer an appealing alternative to traditional running-based high-intensity interval training (HIIT), which is well-established for its efficacy in improving player’s endurance performance, repeated sprint ability (RSA), change of direction, and achieving maximal speed in linear sprints, depending on the specific HIIT protocols employed (Clemente et al., 2021c).

Although the use of SSGs has been supported by original studies and systematic reviews as an effective method for improving endurance performance comparable to HIIT their effectiveness in enhancing change of direction, sprinting, and RSAs is not as evident (Hammami et al., 2018). This may be attributed to the limited stimulus provided in certain locomotor demands, such as high-speed running and sprint distances, due to the restricted space available for achieving high speeds during small and medium SSGs drills (Castagna et al., 2017). Moreover, the heterogeneity of these outcomes influenced by contextual factors that affect the occurrence of events requiring maximal locomotor demands, can also contribute to the lesser effectiveness of SSGs in improving change of direction, sprinting (>30 m), and repeated short sprints (<10 m) (Filipe et al., 2022).

To harness the advantages of both SSGs and HIIT, researchers have explored the potential benefits of combining these training methods in order to elicit different effects on the physical fitness of soccer players (Clemente and Sarmento, 2021). For example, a study by Harrison et al. (2015) compared a combined SSGs + HIIT (short intervals) intervention with a purely SSGs-based intervention. The results showed that players exposed to the combined format significantly increased maximal oxygen uptake compared to those only participating in SSGs. However, no significant differences were found between the groups in terms of linear sprint performance (Harrison et al., 2015). In another study that combined SSGs + HIIT (sprint interval training and repeated sprint training), no significant advantages were observed compared to SSGs alone, while the average outcomes actually favored the SSGs group (Castillo et al., 2021). Additionally, a study comparing two different combined formats (one starting with SSGs and transitioning to HIIT, and the other *vice versa*) demonstrated that both formats led to similar significant improvements in endurance performance, as assessed by the 30–15 Intermittent Fitness Test (Rabbani et al., 2019).

Despite these findings, the current body of research on the combined use of SSGs and HIIT is limited and inconsistent, highlighting the need for further investigation to obtain more robust evidence. Specifically, it is important to analyze whether a combined SSGs + HIIT approach may yield greater benefits than regular training sessions alone. This analysis should focus on the benefits for endurance performance and other important qualities such as linear sprint, change of direction, and RSA, as these are key physical attributes that can be enhanced by both SSGs and HIIT when examined independently. Additionally, considering the cumulative training load over the intervention period, establishing a dose-response relationship may help identify whether the observed adaptations in physical fitness are directly related to the imposed training program or occur independently.

Therefore, the main objectives of the current research were twofold: (i) to compare the effects of a combined SSGs + HIIT intervention *versus* a control group on measures of endurance performance, linear speed, change of direction, and RSA, and (ii) to analyze the dose-response relationship between the accumulated training load over the intervention period and the observed adaptations in male soccer player’s physical fitness.

## Methods

### Study design

The present study employed a randomized two-arm design. Participants were recruited from a single team competing in the first division of the university championship in Taiwan. Prior to the physical fitness assessments conducted at the beginning of the experimental study, participants were randomized using an electronic-based software (Research Randomizer), utilizing a simple randomization process with 1:1 ratio. Allocation concealment was ensured as the random allocation sequence was implemented without prior knowledge of which player would receive which intervention.

### Ethical procedures

All participants were provided with detailed information regarding the study design, potential risks, and benefits, and provided voluntary written informed consent to participate prior to the study commencement. The study was approved by the ethics committee of the University of Taipei (UT-IRB-2020-061). The study was conducted in adherence to the principles outlined in the Declaration of Helsinki.

### Experimental approach

Participants were randomly assigned to either the intervention group or the control group. The intervention group underwent a 4-week training program consisting of additional sessions of SSGs combined with running-based HIIT training. These sessions were conducted twice a week, resulting in a total of eight sessions, with four sessions dedicated to SSGs and four sessions focusing on short-interval running. The control group followed the same field-based training as their counterparts but did not participate in the additional SSGs and running-based HIIT sessions. Physical fitness assessments were conducted at baseline (1 week prior to the intervention) and post-intervention (1 week following the 4-week intervention period). The intervention took place during the first half of the competitive season. During the experimental period, all players participated in four friendly matches.

### Participants

In order to minimize the likelihood of type I statistical errors, the sample size for this study was determined using G Power 3.1.9.4 software. A power of 80% and an alpha value of 0.05 were employed in a two-tailed test to estimate the minimum number of participants needed. Drawing from the study designs of previous research (Rabbani et al., 2019) it was determined that a minimum of 11 participants in the training group would be required to minimize type I errors in the comparisons between interventions. The inclusion criteria for participant selection were as follows: 1) regular participation in soccer training at least three times per week with a minimum duration of 2 h per session, and 2) a minimum of 5 years of training experience in the sport. Exclusion criteria included: 1) any history of severe neuromuscular injury, 2) current lower extremity injury, and 3) neurological disorders. Criteria for participant withdrawal from the study included: 1) failure to attend any assessment sessions, and 2) attendance of less than 75% (less than six out of eight) of the training sessions.

Twenty-one soccer players from the first division of the university championship in Taiwan (classified as Tier 2 in the Participants Classification Framework (McKay et al., 2022), representing the Trained/Developmental level) were recruited and randomly assigned to either the SSGs + HIIT training group (n = 11; age: 17.7 ± 1.7 years; stature: 170.9 ± 5.0 cm; body mass: 61.8 ± 4.7 kg; body mass index: 21.2 ± 1.4 kg/m^2^) or the control group (n = 10; age: 17.7 ± 1.8 years; stature: 170.9 ± 5.0 cm; body mass: 61.8 ± 4.7 kg; body mass index: 21.2 ± 1.4 kg/m^2^). The adherence rate to the experimental group was 93.2%. Additionally, no injuries were reported throughout the duration of the study.

### Training intervention

While the control group continued regular on-field soccer training practice as instructed by the coaching staff, the experimental group received an additional intervention consisting of a combination of SSGs and running-based HIIT. These sessions were conducted by the strength and conditioning coach and were completed prior to the participants’ regular on-field training sessions. The details of the intervention are presented in Table 1.

**TABLE 1:** Description of the experimental intervention.

Week/Session	W1S1	W1S2	W2S1	W2S2	W3S1	W3S2	W4S1	W4S2
Sets	2	2	2	2	3	3	3	3
Reps	6	2	6	2	6	3	8	4
Reps. duration	15 s	90 s	15 s	90 s	15 s	60 s	15 s	60 s
Time between sets	4 min	4 min	4 min	4 min	4 min	4 min	4 min	4 min
Time between reps	15 s	90 s	15 s	90 s	15 s	60 s	15 s	60 s
Exercise	Short HIIT	3v3 SSGs	Short HIIT	2v2 SSGs	Short HIIT	1v1 SSGs	Short HIIT	1v1 SSGs
SSGs description	-	20 × 18 m | 60 m^2^ per player	-	16 × 15 m | 60 m^2^ per player	-	12 × 10 m | 60 m^2^ per player	-	12 × 10 m | 60 m^2^ per player
Intensity at work	90% V_IFT_	-	100% V_IFT_	-	95% V_IFT_	-	100% V_IFT_	-
Intensity between reps	Rest	Rest	Rest	Rest	Rest	Rest	Rest	Rest
Intensity between sets	65% V_IFT_	65% V_IFT_	65% V_IFT_	65% V_IFT_	65% V_IFT_	65% V_IFT_	65% V_IFT_	65% V_IFT_
Total work	6 min	6 min	6 min	6 min	9 min	10 min	12 min	12 min

W, week; m, meters; S, session; Reps, repetitions; VIFT, final velocity at 30–15 Intermittent Fitness test; HIIT, running-based high-intensity interval training; SSGs, small-sided games.

Both the SSGs and running-based HIIT sessions took place on artificial turf and were conducted prior to the participants’ regular on-field training sessions. During the SSGs implemented in this study, goalkeepers were not included, and the primary objective was to maintain ball possession for as long as possible via consecutive successful passing. No specific offside rules were enforced, and no verbal encouragement was provided during the games. Multiple balls were positioned along the boundaries of the pitch to facilitate immediate replacement when a ball went out of bounds.

### Physical fitness assessment

The physical fitness assessments were conducted 1 week prior to the start of the intervention and 1 week following its completion. All assessments took place on the same day of the week, specifically during the first training session of the week, following a 48-h rest period after the latest match. The assessments were scheduled to begin at 4.00 p.m. The environmental conditions during the assessments were maintained at a temperature of 20ºC ± 1.5°C and relative humidity of 65% ± 4%.

Prior to the assessments, a standardized warm-up protocol was performed. This protocol included a 5-min self-paced jogging exercise, followed by approximately 5 minutes of lower limb dynamic stretching exercises. Additionally, specific exercises focusing on jumping and acceleration were performed for approximately 3 min.

The sequence of the physical fitness assessments was as follows: (i) countermovement jump (CMJ), (ii) change-of-direction test, (iii) sprint test, (iv) RSA test, and (v) 30–15 Intermittent Fitness test. A rest period of 5 minutes was provided between each test to ensure adequate recovery.

### Countermovement jump

The CMJ with fixed arms test was employed as a means to evaluate jump height within the scope of this particular investigation. Participants were instructed to assume an upright starting position, then flex their lower extremities and subsequently execute a jump without any pause between these phases. Throughout the jumping motion, participants were specifically directed to maintain extended knees and ensure simultaneous foot contact upon landing. Notably, the participants were instructed to fix their hands on their hips for the entirety of the movement. To mitigate any potential unfamiliarity with the CMJ technique, participants were familiarized with the jump through prior training routines.

Countermovement jump performance was quantified using a two-axis portable force platform (PASCO, Pasport PS-2142, Roseville, USA). The force platform was utilized to measure the vertical displacement achieved during the CMJ. Participants completed three trials, each separated by a 30-s interval. The highest jump height recorded in centimeters was selected as the representative value for subsequent statistical analyses (Anicic et al., 2023).

### Change of direction

The 5–0–5 test, in its original form, was utilized for this study. This test involves accelerating at maximum intensity for a distance of 10 m followed by a 5-m sprint performed at maximal intensity. Subsequently, a 180° change-of-direction (COD) maneuver is executed, followed by another 5-m maximal intensity sprint back to the starting point.

To ensure randomness and fairness, the players were randomly assigned to two groups. Half of the players commenced the trials by braking with their preferred leg at the COD line, while the remaining players initiated the trials by braking with their non-preferred leg. Each player performed three attempts using one leg before switching to the opposite leg for braking at the COD line. A rest period of 2 min was provided between each attempt to allow for adequate recovery. As the test was already a part of the team’s regular assessment routines, the players were familiar with its execution and requirements. Participants completed the test wearing standard soccer boots, which are the footwear used during regular training and matches.

For the starting position, the players began 0.3 m away from the first pair of photocells (Fusion Sport, Coopers Plains, Australia), which were placed 60 cm above the ground and located at the starting line. Participants adopted a staggered stance, consistently placing the same foot in front. The best time achieved from the three trials for each foot was used as the reference measurement (expressed in seconds). From these measurements, three variables were derived: COD time, which represents the time taken to complete the test; COD deficit, which quantifies the difference between the 10-m COD time and the 10-m linear sprint time measured in the separate sprint test and COD asymmetry, that reflects the disparity between the COD times of the player’s best and worst legs (with the best leg being the one with the shorter COD time) (Dos Santos et al., 2019).

### Linear sprint test

The participants completed three trials of the 30-m linear sprint test. A rest period of 2 min was provided between each trial to ensure sufficient recovery. The participants were instructed to start the sprint in a staggered stance position, with their preferred foot in front. Participants were positioned 0.3 m away from the first pair of photocells, which were placed 60 cm above the ground and located at the starting line.

Three pairs of photocells were used for timing: one pair at the starting line (0 m), another pair at the 10-m mark, and a final pair at the finish line (30 m). The participants were specifically instructed to sprint as fast as possible from the starting line to the end of the 30-m track and to decelerate only after crossing the 30-m line. The split times (in seconds) for the 0–10 m and 0–30 m intervals were recorded for each trial. The best trials for the 0–10 m and 0–30 m intervals were selected for further analysis and data treatment (Altmann et al., 2019).

### Repeated sprint ability (RSA)

The RSA protocol consisted of a 20-m shuttle sprint with a 20-s rest interval. The participants performed a total of six repeated sprints as part of the RSA test. To prevent pacing and ensure maximal effort, the participants were not informed of the number of sprints to be performed or the criteria for termination.

To measure the sprint time, a timing gate system (Fusion Sport, Coopers Plains, Australia) was positioned at both the starting and return lines. The players started with their preferred leg and were positioned in a staggered stance position, with their preferred foot in front. This system accurately recorded the time taken for each sprint. The following outcomes were extracted from the test results: mean RSA (the average sprint time across the six sprints performed), total RSA (the sum of the sprint times over the six sprints), best RSA (the shortest sprint time among the six sprints), worst RSA (the longest sprint time among the six sprints), and RSA decrement ([RSA total/(RSA best x number of sprints)] × 100) (Girard et al., 2011).

### 30–15 intermittent fitness test (IFT)

The original 30–15IFT (30–15 Intermittent Fitness Test) was utilized for this study (Buchheit et al., 2009). The test involves performing 30-s shuttle runs with 15-s walking recovery periods. The test takes place on a field measuring 40 m and is divided into three-line zones: A, B, and C. Zone B is located in the middle of the field (20 m). The 30–15IFT commences at an initial speed of 8 km/SSGs and progressively increases by 0.5 km/SSGs at each 30-s stage. The players synchronize the running pace with audio beeps provided during the test. If a player fails to sustain the required pace or does not reach the designated line zone prior to the beep on three consecutive occasions, the test is concluded. The final velocity attained during the 30–15IFT, known as VIFT (final velocity at 30–15IFT), is determined by the speed achieved in the last successfully completed stage. V_IFT_ serves as the primary outcome measure for subsequent data analysis and interpretation.

### Training load monitoring

During the 4-week experimental phase of the study, both the experimental and control group participants utilized the same Global Navigation Satellite Systems (GNSS) equipment (10 Hz, Catapult playertek team, Catapult, Australia). This system incorporated a 3-dimensional accelerometer, a gyroscope, and a digital compass, which sampled data at a rate of 200 Hz. Locomotor and mechanical training demands produced by the players were monitored daily. The GNSS also integrates a heart rate monitor that allows the second-by-second heart rate responses of each player to be recorded. The system also provided a measure of heart rate load (HR load). To ensure consistent placement of the sensor, each player positioned it in the center of their chest using the specially designed elastic band provided by the company. Prior to the study, the reliability and validity of this system had been established through previous validation processes, confirming its accuracy in quantifying the most common locomotor demands.

The following physical outcomes were analyzed for each training session: total distance covered, peak speed registered, distance covered in zone 1 (Z1; 3.00–6.99 km/SSGs), distance covered in zone 2 (Z2; 7.00–10.99 km/SSGs), distance covered in zone 3 (Z3; 11.00–14.99 km/SSGs), distance covered in zone 4 (Z4; 15.00–18.99 km/SSGs), distance covered in zone 5 (Z5; 19.00 km/SSGs), time spent in deceleration zones at various rates (0–1 m/s/s, 1–2 m/s/s, 2–3 m/s/s, >4 m/s/s), number of accelerations and decelerations, maximum deceleration and acceleration experienced, work ratio, power score, impact zones of 3–5 G, distance covered in acceleration zones (0–1 m/s/s, 1–2 m/s/s, 2–3 m/s/s), time spent in acceleration zones (1–2 m/s/s, 2–3 m/s/s), and distance covered in power zones (0–5 SSGs/kg, 15–20 SSGs/kg, 25–30 SSGs/kg, 30–35 SSGs/kg, >50 SSGs/kg).

In addition to the use of sensors for monitoring physical exertion, the rate of perceived exertion (RPE) was also assessed using Foster’s 10-point scale (Foster et al., 2001). This scale allows individuals to subjectively rate the perceived level of exertion during training sessions. To calculate the session-RPE, the score provided at the end of the training session is multiplied by the duration of the session in minutes.

The RPE assessments were conducted individually, approximately 30 min following the completion of each training session. Participants were familiarized with the RPE scale prior to the study as it is commonly employed as part of the team’s regular training/monitoring routines. The RPE data were recorded in an Excel spreadsheet for further analysis.

### Statistical procedures

For statistical analysis, 2-way repeated measures ANOVA with a between and within-subjects design was used for the fitness variables. Prior to the commencement of the analysis, the Shapiro-Wilks normality test was carried out and established the normal distribution of the sample. Moreover, the Box’s test assumption for the equality of variance-covariance matrices of difference scores between groups was achieved.

To present the findings, mean values were reported along with their corresponding standard deviations (mean ± SD). Descriptive statistics were conducted for all variables to provide a comprehensive characteristic summary. In addition, Cohen’s d effect size analysis was employed to examine the magnitude of differences within the study sample which was evaluated using the Hopkins scale as follows: 0–0.2, trivial; 0.2–0.6, small; 0.6–1.2, moderate; 1.2–2.0, large; >2.0, very large (Cohen, 1988). To determine statistical significance, the level of significance was set at *p* < 0.05. SPSS Statistical Software v.24.0, Spyder v3.6.6, Python (v3.7) IDE, and its associated scikit-learn libraries, coupled with Jamovi Version 2.3 (The Jamovi Project 2022) were utilized for the statistical analyzes, which facilitated data processing and computations.

### Identifying relevant GPS variables

Extremely Randomized Trees (ERT) is a tree-based ensemble technique used for supervised classification and regression problems. It is a variant of Random Forests that constructs decision trees by utilizing random thresholds for each feature and selecting the most appropriate feature among a random subset of features at each node. Numerous studies have demonstrated that ERT yields more precise results than other approaches such as Support Vector Machines (SVM) and Random Forests (Geurts and Wehenkel, 2000; Geurts et al., 2006). Extremely Randomized Trees is ideal over other methods for feature extraction due to its capability of handling high-dimensional data consisting of multiple features. Due to the high dimensionality of the GPS dataset in the present investigation, ERT was employed to identify the essential GPS parameters that are relevant to the groups of examined players.

### Cluster analysis for defining player load

K-means clustering is a widely adopted unsupervised machine learning algorithm that is utilized to classify data points into clusters or groups according to their similarity The algorithm partitions the data into k clusters specified by the user. Each data point is then assigned to the closest cluster center and updated the cluster centers are based on the mean of the data points allocated to each cluster (Muazu Musa et al., 2019). The algorithm proceeds with this iterative process until a state of convergence is attained. In the context of this study, k-means clustering was employed to categorize the cumulative load that the players were subjected to during the intervention period.

### Model development for understanding dose-response relationships among the GPS study variables

A dose-response-based model of performance was created by using a multivariate binary logistic regression. The impact of each variable on the group of player’s performances was determined, and the magnitude of the changes in the variables was predicted. In this analysis, the independent variables were the variables identified via the ERT selection method, while the group of players, i.e., control and experimental, served as the dependent variables. The levels of players’ load identified through the k-mean clustering were introduced to the model as a covariate to test the predictive ability of the model while accounting for the effect of different loads exposed to the players. This technique is useful in identifying the most significant variables that could differentiate the group of players concerning the specific training they receive during the intervention period. The Forward stepwise selection method (Likelihood Ratio) was employed to analyze the data. The results were reported in terms of odds ratios (OR) and 95% confidence intervals (CI). Nagelkerke’s *R* ^2^ was used to evaluate the model’s explanatory power, with the effect size interpreted as small (0.02–0.13), medium (0.13–0.26), and large (>0.26). The model’s goodness of fit was assessed using the Hosmer-Lemeshow test, and the discriminant capacity of the model was evaluated using the area under the curve (AUC) and the Receiver Operating Characteristic (ROC) curve, which was generated using the predicted probabilities for each variable.

## Results

### Analysis of between subjects-effects

No statistical significance differences were observed between groups for any of the examined fitness variables. However, despite the lack of significant difference between the two groups, it was observed that the experimental group (SSGs + HIIT) recorded a noteworthy improvement in certain fitness levels during post-intervention compared to the control group (Table 2). These fitness variables consist of asymmetry index (% change = 46.9, Cohen’s d = 0.33), mean RSA (% change = 3.8, Cohen’s d = 0.55), worse RSA (% change = 3.7, Cohen’s d = 0.43), CMJ (% change = −2.7, Cohen’s d = 0.35) and the 30–15IFT (% change = 8.8, Cohen’s d = 0.41).

**TABLE 2:** Inferential and descriptive statistics of the fitness variables.

Variables	SSGs + HIIT (n = 11)			Control group (n = 10)			Between group (baseline)	Between group (post intervention)
	Baseline	Post-intervention	Within group	Baseline	Post-intervention	Within group		
Resting heart rate (bpm)	70.818 ± 13.636	75.909 ± 10.568	*F* = 1.20; *p* = 0.299; η^2^_*p*_ = 0.107	71.39 ± 214	76.7 ± 5.945^a^	*F* = 6.83; *p* < 0.05*; η^2^_*p*_ = 0.431	t = 0.093; *p* = 0.926; d = 0.041	t = 0.208; *p* = 0.837; d = 0.091
% Change	+7.2		N/A	+7.4		N/A	N/A	N/A
Sprint 10 m (s)	1.824 ± 0.099	1.785 ± 0.0688	*F* = 1.57; *p* = 0.239; η^2^_*p*_ = 0.136	1.833 ± 0.068	1.825 ± 0.075	F = 1.10; *p* = 0.321; η^2^ *p* = 0.109	t = 0.248; *p* = 0.807; d = 0.108	t = 0.777; *p* = 0.447; d = 0.339
% Change	−2.1		N/A	−0.44		N/A	N/A	N/A
30-m linear sprint (s)	4.293 ± 0.137	4.299 ± 0.137	*F* = 5.21; *p* = 1.000; η^2^_*p*_ = 0.001	4.346 ± 0.119	4.336 ± 0.210	*F* = 0.043; *p* = 0.839; η^2^_p_ = 0.005	t = 0.941; *p* = 0.359; d = 0.411	t = 0.536; *p* = 0.598; d = 0.234
% Change	+0.14		N/A	−0.23		N/A	N/A	N/A
Left COD 5-0–5 test (s)	2.239 ± 0.053	2.330 ± 0.097^aa^	*F* = 29.4; *p* < 0.001*; η^2^_p_ = 0.746	2.236 ± 0.101	2.374 ± 0.136^aa^	*F* = 48.2; *p* < 0.001*; η^2^_p_ = 0.843	t = - 0.088; *p* = 0.930; d = - 0.038	t = 0.858; *p* = 0.402; d = 0.375
% Change	+4.1		N/A	+6.2		N/A	N/A	N/A
Right COD 5-0–5 test (s)	2.263 ± 0.0618	2.336 ± 0.111^a^	*F* = 6.37; *p* < 0.05*; η^2^_*p*_ = 0.389	2.298 ± 0.068	2.378 ± 0.109^a^	*F* = 6.74; *p* < 0.05*; η^2^_p_ = 0.428	t = 1.238; *p* = 0.231; d = 0.541	t = 0.863; *p* = 0.399; d = 0.377
% Change	+3.2		N/A	+3.5		N/A	N/A	N/A
Best COD time (s)	2.230 ± 0.055	2.301 ± 0.078^aa^	*F* = 26.5; *p* < 0.001*; η^2^_*p*_ = 0.726	2.230 ± 0.09	2.353 ± 0.130^aa^	*F* = 33.0; *p* < 0.001*; η^2^_*p*_ = 0.786	t = 0.001; *p* = 1.000; d = 0.001	t = 1.125; *p* = 0.275; d = 0.491
% Change	+3.2		N/A	+5.5		N/A	N/A	N/A
Asymmetry index COD (s)	1.889 ± 1.506	2.775 ± 2.422	*F* = 0.941; *p* = 0.0941; η^2^_*p*_ = 0.086	3.397 ± 2.620	2.017 ± 2.123^a^	*F* = 13.7; *p* < 0.005*; η^2^_*p*_ = 0.603	t = - 1.637; *p* = 0.118; d = - 0.715	t = 0.758; *p* = 0.457; d = 0.331
% Change	+46.9		N/A	−40.6		N/A	N/A	N/A
COD deficit (s)	0.406 ± 0.117	0.516 ± 0.090^a^	*F* = 8.26; *p* < 0.01*; η^2^_*p*_ = 0.452	0.397 ± 0.089	0.544 ± 0.130^aa^	*F* = 25.0; *p* < 0.001*; η^2^_*p*_ = 0.735	t = - 0.206; *p* = 0.841; d = - 0.089	t = 0.569; *p* = 0.576; d = 0.249
% Change	+27.1		N/A	+37.0		N/A	N/A	N/A
Mean repeated sprint ability (s)	7.937 ± 0.292	8.239 ± 0.458^a^	*F* = 7.57; *p* < 0.05*; η^2^_*p*_ = 0.431	7.835 ± 0.383	7.942 ± 0.624	*F* = 1.04; *p* = 0.335; η^2^_*p*_ = 0.103	t = −0.689; *p* = 0.499; d = - 0.301	t = −1.253; *p* = 0.225; d = −0.547
% Change	+3.8		N/A	+1.4		N/A	N/A	N/A
Total RSA (s)	46.911 ± 3.105	48.714 ± 4.020^a^	*F* = 7.45; *p* < 0.05*; η^2^_*p*_ = 0.427	45.511 ± 5.872	46.874 ± 4.675	*F* = 0.611; *p* = 0.454; η^2^_*p*_ = 0.064	t = −0.692; *p* = 0.497 days = - 0.302	t = −0.969; *p* = 0.345; d = −0.423
% Change	+3.8		N/A	+3.0		N/A	N/A	N/A
Best RSA (s)	7.494 ± 0.365	7.857 ± 0.404^a^	*F* = 7.65; *p* < 0.05*; η^2^_*p*_ = 0.434	7.419 ± 0.247	7.565 ± 0.488	*F* = 1.72; *p* = 0.222; η^2^_*p*_ = 0.161	t = −0.541; *p* = 0.594 days = -0.236	t = −1.499; *p* = 0.150; d = −0.655
% Change	+4.8		N/A	+2.0		N/A	N/A	N/A
Worst RSA (s)	8.311 ± 0.295	8.617 ± 0.555	*F* = 3.94; *p* = 0.075; η^2^_*p*_ = 0.283	8.139 ± 0.430	8.321 ± 0.801	*F* = 1.47; *p* = 0.256; η^2^_*p*_ = 0.141	t = −1.076; *p* = 0.295 days = - 0.470	t = −0.992; *p* = 0.333; d = −0.433
% Change	+3.7		N/A	+2.2		N/A	N/A	N/A
RSA decrement (s)	4.380 ± 5.752	3.294 ± 6.136	*F* = 0.644; *p* = 0.441; η^2^_*p*_ = 0.060	2.111 ± 11.760	3.169 ± 5.952	*F* = 0.061; *p* = 0.809; η^2^ *p* = 0.007	t = 0.570; *p* = 0.575 days = - 0.249	t = −0.047; *p* = 0.963; d = −0.020
% Change	−24.8		N/A	+50.1		N/A	N/A	N/A
CMJ (cm)	40.889 ± 5.804	39.786 ± 4.760	*F* = 1.44; *p* = 0.258; η^2^_*p*_ = 0.126	40.209 ± 5.093	37.809 ± 6.343	*F* = 3.91; *p* = 0.079; η^2^_*p*_ = 0.303	t = -0.284; *p* = 0.779 days = − 0.124	t = −0.813; *p* = 0.426; d = - 0.355
% Change	−2.7		N/A	−6.0		N/A	N/A	N/A
30–15 IFT test (km)	18.636 ± 0.977	20.273 ± 1.232^aa^	*F* = 21.0; *p* < 0.001*; η^2^_*p*_ = 0.677	19.000 ± 1.269	19.700 ± 1.531	*F* = 4.85; *p* = 0.055; η^2^_*p*_ = 0.350	t = 0.739; *p* = 0.468 days = - 0.323	t = −0.948; *p* = 0.355; d = −0.414
% Change	+8.8		N/A	+3.7		N/A	N/A	N/A

Notes: a: Significant interactions over time (*p* < 0.05).

aa: Significant interactions over time (*p* < 0.001).

*: Significant different within player variation.

N/A: not applicable.

### Analysis of within subject-effects and time interactions

As shown in Table 2, significant interactions were observed across 10 fitness variables. The main effect of time on resting heart rate was significant in the control group (*p* < 0.05) but not in the experimental group (*p* > 0.05). For left change of direction, significant effects were found across all groups (*p* < 0.001) and within the experimental group (*p* < 0.001). Significant interactions of time and within-group performance were noted for the right change of direction across groups (*p* < 0.05) and for the best change of direction (*p* < 0.001). The asymmetry index showed significant changes in the control group (*p* < 0.05) but not in the experimental group (*p* > 0.05). Main effects of time and within-group interactions for COD deficit were significant across all groups (*p* < 0.001). For the experimental group, changes in mean, total, and best RSA were significant (*p* < 0.05), while no significant changes were observed in the control group (*p* > 0.05). Finally, changes in the 30–15IFT were significant for the experimental group (*p* < 0.001) but not for the control group (*p* > 0.05).

**FIGURE 1:**
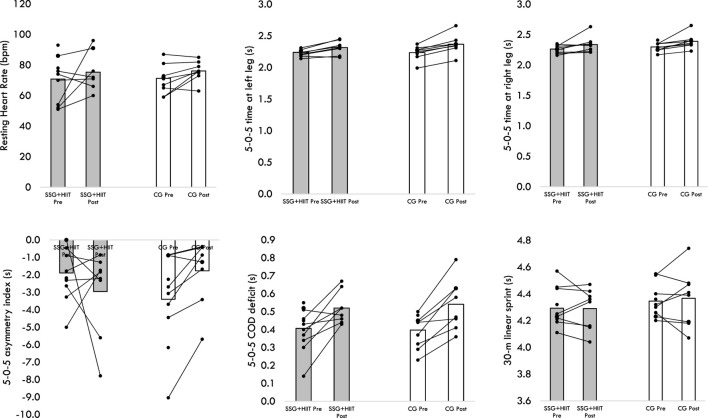
Variations of both groups in resting heart rate, 30-m linear sprint and 5-0-5 test performance.

**FIGURE 2:**
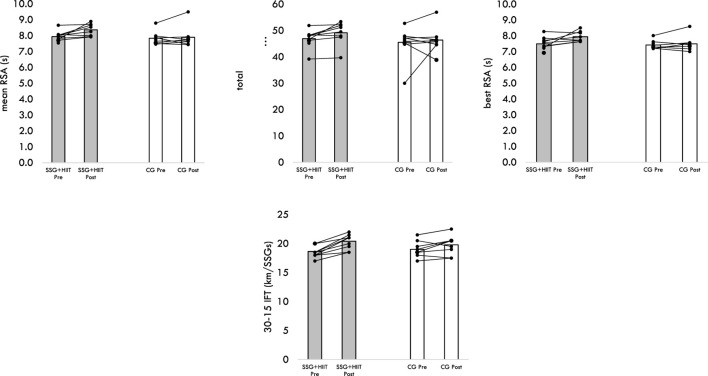
Variations of both groups in repeated sprint ability test and the 30-15 Intermittent Fitness test performances.

Figures 1, 2 display the variation of groups for the different outcomes, considering the within-player variation.

**FIGURE 3:**
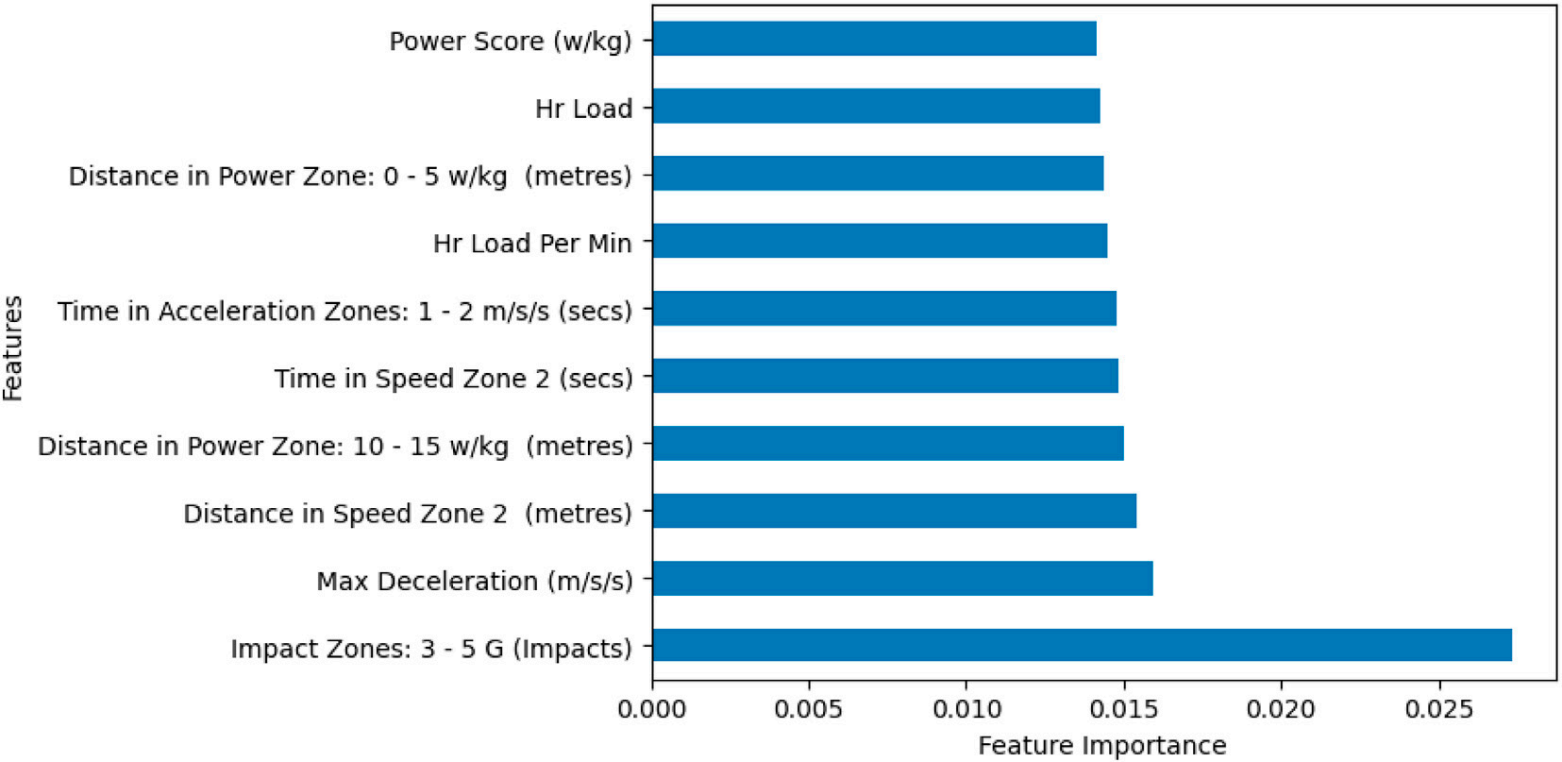
Relevant and essential GPS variables results based on extremely randomized tree analysis.

Figure 3 illustrates the ERT technique results, identifying the 10 most important variables out of 93 initially gathered. These variables are crucial for player performance across both groups and were used to develop the logistic regression model. Descriptive statistics for the remaining non-essential variables are available in Supplementary Appendix S1.

**FIGURE 4:**
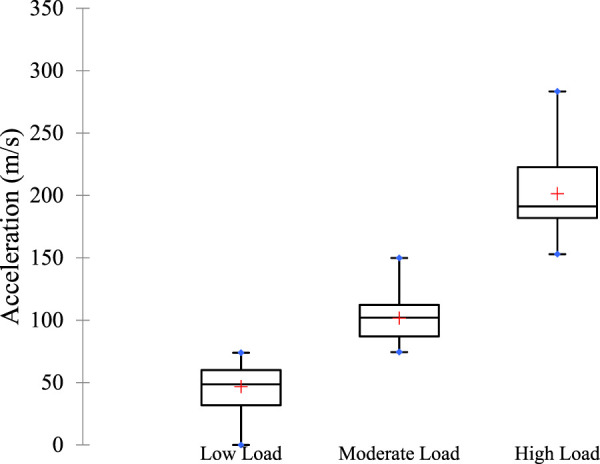
Classification of player load.

Figure 4 shows the k-means clustering analysis of players’ load, identifying three categories: low, moderate, and high. The mean loads for these categories were 46.71, 101.66, and 201.36, respectively. These load levels were used as covariates in model building.

Table 3 shows the multivariable binary logistic regression model assessing GPS variables for predicting changes between the two player groups. The model had good fit (Hosmer-Lemeshow >.05), classification accuracy (68%), and discriminant capacity (AUC 76%). It explained 26% of the variance (Negelkerke *R* ^2^ = 0.26). Seven key variables were significant in predicting group membership (*p* < 0.05): heart rate load per minute, time in speed zone 2 (secs), impact zones 3-5G, maximum deceleration, time in acceleration zones 1–2 m/s^2^ (secs), distance in power zone 0–5 SSGs/kg (m), and distance in power zone 10–15 SSGs/kg (m).

**TABLE 3:** Logistic regression model parameters for determining the predictive variables.

Variables	*B*	Se	Z	*p*	Odds ratio	95% CI	
						Lower	Upper
Intercept	−0.091	1.003	−0.091	0.928	0.913	0.128	6.522
Hr Load	0.004	0.007	0.651	0.515	1.004	0.991	1.018
Hr Load Per Min	0.471	0.188	2.504	0.012*	1.602	1.108	2.316
Time in Speed Zone 2 (secs)	−0.004	0.001	−2.474	0.013*	0.996	0.994	0.999
Impact Zones: 3–5 G (Impacts)	−0.013	0.002	−5.363	0.001*	0.987	0.983	0.992
Max Deceleration (m/s/s)	−0.107	0.052	−2.072	0.038*	0.898	0.812	0.994
Power Score (SSGs/kg)	0.019	0.071	0.269	0.788	1.019	0.887	1.172
Distance in Speed Zone 2 (metres)	0.001	0.001	1.318	0.188	1.001	0.999	1.003
Time in Acceleration Zones: 1–2 m/s/s (secs)	−0.050	0.006	−8.218	0.001*	0.951	0.940	0.963
Distance in Power Zone: 0–5 SSGs/kg (metres)	0.005	0.001	7.121	0.001*	1.005	1.004	1.007
Distance in Power Zone: 10–15 SSGs/kg (metres)	0.004	0.001	3.181	0.001*	1.004	1.001	1.006
Player Load							
Low Load – High Load	0.056	0.884	0.064	0.949	1.058	0.187	5.986
Moderate Load – High Load	0.261	0.721	0.362	0.717	1.298	0.316	5.334

Note. *Sig; Nagelkerke *R* ^2^ = 0.262; Hosmer Lemeshow (*p* = 0.810); Classification Accuracy = 0.682; AUC, 0.759.

Odds ratios showed that the experimental group had a 60% higher chance of increased heart rate load per minute (OR 1.602) and a 0.4% lower chance of spending time in speed zone 2 s (OR 0.996). The control group had a 1.3% higher probability of decreased impact zones 3-5G (OR 0.987) and a 10% higher chance of reduced maximum deceleration (OR 0.989). For each unit increase in time in acceleration zones 1–2 m/s^2^, the control group had a 4.9% higher likelihood of decreased maximum acceleration. The experimental group was 0.5% and 0.4% more likely to cover additional distances in power zones 0–5 and 10–15 SSGs/kg, respectively (ORs 1.005 and 1.004). Player load changes from low to high or moderate to high did not significantly affect group performance (*p* > 0.05).

## Discussion

The present study aimed to investigate the effects of a 4-week small-sided games (SSGs) intervention combined with running-based high-intensity interval training (HIIT) on the physical fitness of collegiate male soccer players. The main findings showed that the SSGs intervention combined with HIIT had a positive impact on the physical fitness of collegiate male soccer players. However, the improvements observed across various fitness variables were generally small, and in some cases, such as the countermovement jump (CMJ), performance slightly decreased in both groups. These modest improvements, while not reaching statistical significance, were associated with small to moderate effect sizes, indicating that the combined training approach could still be potentially beneficial for enhancing athletic performance among collegiate male soccer players, albeit with some limitations.

Firstly, the intervention group, that underwent the combined SSGs and HIIT training, showed noteworthy improvements in overall physical fitness measures compared to the control group. Specifically, improvements were observed in the asymmetry index, mean RSA, worst RSA, CMJ, and the 30–15IFT, with small to moderate effect sizes. Despite the decrease in CMJ performance, the observed improvements in other areas, particularly in RSA and 30–15IFT, suggest that the combined SSGs and HIIT training might still have positively impacted aspects of physical fitness relevant to soccer performance. The combination of SSGs and HIIT interventions may have contributed to these improvements in physical fitness (Arslan et al., 2021). Small-sided games are known to stimulate acute responses above 85% of maximal heart rate and offer an effective method for enhancing cardiovascular fitness and specific neuromuscular aspects crucial for endurance performance (Los Arcos et al., 2015). The intermittent nature of high-intensity efforts in SSGs promotes enhanced elastic energy storage and release during subsequent jumps, leading to improved CMJ performance (Clemente et al., 2021b). However, the observed decrease in CMJ might be explained by factors such as accumulated fatigue over the intervention period or the focus of the training on endurance and agility rather than explosive power (Gathercole et al., 2015). High-intensity interval training has also been shown to elicit favorable adaptations in endurance performance, with increased mitochondrial content and oxygen consumption in muscle tissue leading to improved aerobic responses (Atakan et al., 2021). The combination of both SSGs and HIIT interventions in the present study likely synergized these effects, contributing to the observed improvements in RSA, and 30–15IFT (Rabbani et al., 2019).

The experimental group showed higher likelihoods of increased heart rate load per minute, reduced time in speed zone 2, elevated impact zones, greater maximum deceleration, decreased time in acceleration zones, and greater distances covered in power zones compared to the control group. This is a typical example of the dose-response relationship, referring to the relationship between the dose of the intervention and its effect on different outcomes (Impellizzeri et al., 2023). For instance, a previous study explored the links between accumulated external load and changes in body composition, isokinetic strength, and aerobic capacity in professional soccer players over a 10-week period (Clemente et al., 2019). The authors found significant positive correlations between sprinting distance and percentage differences in body mass, heart rate maximum (HRmax), and specific strength ratios. These results underpin the importance of monitoring external load to enhance players’ physical performance and mitigate injury risks in soccer, indicating dose-response relationships using external load variables.

The analysis of external load measures revealed notable differences between the intervention and control groups. The experimental group exhibited higher heart rate load per minute and increased distances covered in power zones (0–5 SSGs/kg and 10–15 SSGs/kg), alongside elevated impact zones and maximum deceleration, compared to the control group. Specifically, the odds ratios for heart rate load per minute (OR 1.602) and distances in power zones (OR 1.005 and 1.004) indicated a greater likelihood of increased load and performance in the experimental group. These findings underscore the impact of the combined SSGs and HIIT approach on enhancing external load variables, which are critical for assessing training intensity and player performance (Rabbani et al., 2019). However, shifts in load categories from low to high or moderate to high did not significantly influence changes in physical fitness measures, suggesting that while external load is an important factor, its direct relationship with performance improvements over a short intervention period may be complex and warrant further investigation.

Notwithstanding the findings of our study, further research with larger sample size and potentially longer intervention periods may be warranted to strengthen the evidence and draw more robust conclusions. Additionally, future studies may consider examining the effects of the intervention on other performance-related outcomes and exploring potential differences based on individual player characteristics, such as skill levels and training experience. Still, the present study contributes to the understanding of the effectiveness of the combined SSGs and HIIT training intervention in collegiate male soccer players. The findings demonstrate the potential of this approach to enhance physical fitness, which has implications for optimizing training strategies and improving athletic performance in the sport of soccer.

## Conclusion

In conclusion, the 4-week intervention combining SSGs with running-based HIIT did not produce statistically significant improvements in most physical fitness variables when compared to the control group. Although the experimental group showed positive trends with small to moderate effect sizes in variables such as the RSA and the 30–15IFT, these changes did not reach statistical significance. The modest improvements observed in the experimental group suggest that while the combined SSGs and HIIT approach has potential, its current implementation may not yield substantial changes in physical fitness over a 4-week period. Notably, some variables, such as the CMJ, even exhibited decreases, indicating that the intervention’s impact may be limited or inconsistent.

## Data Availability

The raw data supporting the conclusions of this article will be made available by the authors, without undue reservation.
