# Supplementary material for: Assessing physical fitness adaptations in collegiate male soccer players through training load parameters: a two-arm randomized study on combined small-sided games and running-based high-intensity interval training
Source: Front Physiol. 2024 Sep 11;15:1466386. doi: 10.3389/fphys.2024.1466386 (PMC11440478; doi:10.3389/fphys.2024.1466386)
Supplement: Supplementary file 1 [file Table1.docx]

Appendix 1. Descriptive statistics of the remaining variables of the logistic regression that were not found to be essential

| Descriptives | | | | | | | | | | | |
| --- | --- | --- | --- | --- | --- | --- | --- | --- | --- | --- | --- |
|  | | **Group** | | **Mean** | | **SD** | | **Minimum** | | **Maximum** | |
| Distance (metres) |  | Control Group |  | 1405.15549 |  | 1092.3848 |  | 0.00000 |  | 5960.48 |  |
|  |  | SSGs+HIIT-Group |  | 1551.57916 |  | 1067.3263 |  | 0.00000 |  | 5536.38 |  |
| Sprints |  | Control Group |  | 1.06715 |  | 2.7299 |  | 0 |  | 17 |  |
|  |  | SSGs+HIIT-Group |  | 1.55078 |  | 3.6027 |  | 0 |  | 23 |  |
| Sprint Distance (m) |  | Control Group |  | 37.84830 |  | 83.0127 |  | 0.00000 |  | 503.44 |  |
|  |  | SSGs+HIIT-Group |  | 63.42804 |  | 149.3781 |  | 0.00000 |  | 1010.72 |  |
| Accelerations |  | Control Group |  | 15.27578 |  | 14.2329 |  | 0 |  | 98 |  |
|  |  | SSGs+HIIT-Group |  | 16.27344 |  | 13.7307 |  | 0 |  | 67 |  |
| Decelerations |  | Control Group |  | 14.27818 |  | 15.4169 |  | 0 |  | 97 |  |
|  |  | SSGs+HIIT-Group |  | 13.19727 |  | 14.8327 |  | 0 |  | 94 |  |
| Power Plays |  | Control Group |  | 5.75779 |  | 9.5712 |  | 0 |  | 61 |  |
|  |  | SSGs+HIIT-Group |  | 5.97070 |  | 8.1896 |  | 0 |  | 44 |  |
| Hr Load |  | Control Group |  | 33.50913 |  | 47.0856 |  | 0.00000 |  | 377.56 |  |
|  |  | SSGs+HIIT-Group |  | 39.64716 |  | 45.5583 |  | 0.00000 |  | 312.08 |  |
| Energy (kcal) |  | Control Group |  | 163.04255 |  | 130.1702 |  | 0.00000 |  | 779.48 |  |
|  |  | SSGs+HIIT-Group |  | 178.47240 |  | 127.5341 |  | 0.00000 |  | 669.23 |  |
| Player Load |  | Control Group |  | 77.16382 |  | 46.8119 |  | 0.00250 |  | 283.35 |  |
|  |  | SSGs+HIIT-Group |  | 80.65354 |  | 46.8630 |  | 0.00000 |  | 248.77 |  |
| Top Speed (km/SSGs) |  | Control Group |  | 18.90241 |  | 5.2831 |  | 1.08000 |  | 31.84 |  |
|  |  | SSGs+HIIT-Group |  | 19.76574 |  | 5.0826 |  | 0.00000 |  | 31.60 |  |
| Max Acceleration (m/s/s) |  | Control Group |  | 4.48862 |  | 1.2514 |  | 0.00000 |  | 7.11 |  |
|  |  | SSGs+HIIT-Group |  | 4.57579 |  | 1.2926 |  | 0.00000 |  | 6.85 |  |
| Max Deceleration (m/s/s) |  | Control Group |  | 4.50399 |  | 1.6249 |  | 0.00000 |  | 8.49 |  |
|  |  | SSGs+HIIT-Group |  | 4.00955 |  | 2.1815 |  | 0.00000 |  | 8.67 |  |
| Work Ratio |  | Control Group |  | 21.36714 |  | 15.4141 |  | 0.00000 |  | 87.56 |  |
|  |  | SSGs+HIIT-Group |  | 25.56467 |  | 18.3615 |  | 0.00000 |  | 91.12 |  |
| Player Load Per Min |  | Control Group |  | 3.16894 |  | 1.0807 |  | 0.00000 |  | 7.02 |  |
|  |  | SSGs+HIIT-Group |  | 3.50496 |  | 1.3871 |  | 0.00000 |  | 8.04 |  |
| Hr Load Per Min |  | Control Group |  | 1.25592 |  | 1.2147 |  | 0.00000 |  | 8.37 |  |
|  |  | SSGs+HIIT-Group |  | 1.56589 |  | 1.3823 |  | 0.00000 |  | 6.87 |  |
| Power Score (SSGs/kg) |  | Control Group |  | 4.33967 |  | 1.9653 |  | 0.00000 |  | 10.84 |  |
|  |  | SSGs+HIIT-Group |  | 5.16868 |  | 2.7592 |  | 0.00000 |  | 13.94 |  |
| Distance in Speed Zone 1 (metres) |  | Control Group |  | 827.13275 |  | 553.8589 |  | 0.00000 |  | 2782.87 |  |
|  |  | SSGs+HIIT-Group |  | 852.02086 |  | 590.1418 |  | 0.00000 |  | 2754.03 |  |
| Distance in Speed Zone 2 (metres) |  | Control Group |  | 416.35929 |  | 489.8850 |  | 0.00000 |  | 3048.41 |  |
|  |  | SSGs+HIIT-Group |  | 478.74458 |  | 495.3261 |  | 0.00000 |  | 1904.76 |  |
| Distance in Speed Zone 3 (metres) |  | Control Group |  | 123.81513 |  | 237.2617 |  | 0.00000 |  | 1863.83 |  |
|  |  | SSGs+HIIT-Group |  | 157.38571 |  | 272.5699 |  | 0.00000 |  | 2457.06 |  |
| Distance in Speed Zone 4 (metres) |  | Control Group |  | 33.44584 |  | 69.9472 |  | 0.00000 |  | 432.97 |  |
|  |  | SSGs+HIIT-Group |  | 58.12709 |  | 142.3675 |  | 0.00000 |  | 1008.62 |  |
| Distance in Speed Zone 5 (metres) |  | Control Group |  | 4.40248 |  | 16.0520 |  | 0.00000 |  | 129.51 |  |
|  |  | SSGs+HIIT-Group |  | 5.30094 |  | 15.9567 |  | 0.00000 |  | 106.55 |  |
| Time in Speed Zone 1 (secs) |  | Control Group |  | 1222.22302 |  | 500.3381 |  | 0.00000 |  | 2741.90 |  |
|  |  | SSGs+HIIT-Group |  | 1189.22969 |  | 595.2828 |  | 0.00000 |  | 2743.40 |  |
| Time in Speed Zone 2 (secs) |  | Control Group |  | 177.60216 |  | 243.6293 |  | 0.00000 |  | 1779.20 |  |
|  |  | SSGs+HIIT-Group |  | 170.10723 |  | 184.2135 |  | 0.00000 |  | 869.50 |  |
| Time in Speed Zone 3 (secs) |  | Control Group |  | 29.29305 |  | 60.4392 |  | 0.00000 |  | 501.40 |  |
|  |  | SSGs+HIIT-Group |  | 35.29941 |  | 64.4620 |  | 0.00000 |  | 633.40 |  |
| Time in Speed Zone 4 (secs) |  | Control Group |  | 5.66499 |  | 11.7341 |  | 0.00000 |  | 71.20 |  |
|  |  | SSGs+HIIT-Group |  | 9.87285 |  | 24.3218 |  | 0.00000 |  | 171.50 |  |
| Time in Speed Zone 5 (secs) |  | Control Group |  | 0.58825 |  | 2.1195 |  | 0.00000 |  | 16.60 |  |
|  |  | SSGs+HIIT-Group |  | 0.71035 |  | 2.1256 |  | 0.00000 |  | 13.90 |  |
| Impact Zones: 3 - 5 G (Impacts) |  | Control Group |  | 50.22062 |  | 54.3158 |  | 0 |  | 489 |  |
|  |  | SSGs+HIIT-Group |  | 38.99023 |  | 42.4234 |  | 0 |  | 404 |  |
| Impact Zones: 5 - 10 G (Impacts) |  | Control Group |  | 0.61871 |  | 1.3871 |  | 0 |  | 11 |  |
|  |  | SSGs+HIIT-Group |  | 0.60938 |  | 1.3605 |  | 0 |  | 14 |  |
| Impact Zones: 10 - 15 G (Impacts) |  | Control Group |  | 0.01439 |  | 0.2075 |  | 0 |  | 4 |  |
|  |  | SSGs+HIIT-Group |  | 0.00195 |  | 0.0442 |  | 0 |  | 1 |  |
| Impact Zones: 15 - 20 G (Impacts) |  | Control Group |  | 0.00000 |  | 0.0000 |  | 0 |  | 0 |  |
|  |  | SSGs+HIIT-Group |  | 0.00000 |  | 0.0000 |  | 0 |  | 0 |  |
| Impact Zones: > 20 G (Impacts) |  | Control Group |  | 0.00000 |  | 0.0000 |  | 0 |  | 0 |  |
|  |  | SSGs+HIIT-Group |  | 0.00000 |  | 0.0000 |  | 0 |  | 0 |  |
| Power Play Duration Zones: 0 - 2.5 s (Power Plays) |  | Control Group |  | 0.00000 |  | 0.0000 |  | 0 |  | 0 |  |
|  |  | SSGs+HIIT-Group |  | 0.00000 |  | 0.0000 |  | 0 |  | 0 |  |
| Power Play Duration Zones: 2.5 - 5 s (Power Plays) |  | Control Group |  | 4.39329 |  | 6.2206 |  | 0 |  | 36 |  |
|  |  | SSGs+HIIT-Group |  | 3.66797 |  | 5.0916 |  | 0 |  | 27 |  |
| Power Play Duration Zones: 5 - 7.5 s (Power Plays) |  | Control Group |  | 1.06475 |  | 2.8281 |  | 0 |  | 16 |  |
|  |  | SSGs+HIIT-Group |  | 0.90820 |  | 2.3419 |  | 0 |  | 15 |  |
| Power Play Duration Zones: 7.5 - 10 s (Power Plays) |  | Control Group |  | 0.23741 |  | 0.8457 |  | 0 |  | 6 |  |
|  |  | SSGs+HIIT-Group |  | 0.21094 |  | 0.8408 |  | 0 |  | 5 |  |
| Power Play Duration Zones: > 10 s (Power Plays) |  | Control Group |  | 0.06235 |  | 0.3746 |  | 0 |  | 3 |  |
|  |  | SSGs+HIIT-Group |  | 1.18359 |  | 4.2146 |  | 0 |  | 23 |  |
| Distance in Deceleration Zones: 0 - 1 m/s/s (metres) |  | Control Group |  | 574.24489 |  | 416.0431 |  | 0.00000 |  | 2225.90 |  |
|  |  | SSGs+HIIT-Group |  | 662.50424 |  | 443.5019 |  | 0.00000 |  | 2121.87 |  |
| Distance in Deceleration Zones: 1 - 2 m/s/s (metres) |  | Control Group |  | 118.45843 |  | 100.6638 |  | 0.00000 |  | 575.21 |  |
|  |  | SSGs+HIIT-Group |  | 112.61132 |  | 89.2764 |  | 0.00000 |  | 475.71 |  |
| Distance in Deceleration Zones: 2 - 3 m/s/s (metres) |  | Control Group |  | 35.02378 |  | 33.2387 |  | 0.00000 |  | 212.29 |  |
|  |  | SSGs+HIIT-Group |  | 31.67295 |  | 30.6522 |  | 0.00000 |  | 167.77 |  |
| Distance in Deceleration Zones: 3 - 4 m/s/s (metres) |  | Control Group |  | 11.10926 |  | 14.1154 |  | 0.00000 |  | 103.54 |  |
|  |  | SSGs+HIIT-Group |  | 9.94899 |  | 12.4358 |  | 0.00000 |  | 89.49 |  |
| Distance in Deceleration Zones: > 4 m/s/s (metres) |  | Control Group |  | 4.17275 |  | 7.4349 |  | 0.00000 |  | 63.00 |  |
|  |  | SSGs+HIIT-Group |  | 3.56542 |  | 5.9731 |  | 0.00000 |  | 37.47 |  |
| Time in Deceleration Zones: 0 - 1 m/s/s (secs) |  | Control Group |  | 596.30887 |  | 243.9200 |  | 0.00000 |  | 1274.00 |  |
|  |  | SSGs+HIIT-Group |  | 598.71641 |  | 270.7131 |  | 0.00000 |  | 1233.00 |  |
| Time in Deceleration Zones: 1 - 2 m/s/s (secs) |  | Control Group |  | 62.48177 |  | 38.6310 |  | 0.00000 |  | 215.90 |  |
|  |  | SSGs+HIIT-Group |  | 57.84941 |  | 37.6365 |  | 0.00000 |  | 192.30 |  |
| Time in Deceleration Zones: 2 - 3 m/s/s (secs) |  | Control Group |  | 14.04868 |  | 11.1663 |  | 0.00000 |  | 71.80 |  |
|  |  | SSGs+HIIT-Group |  | 12.80801 |  | 11.3506 |  | 0.00000 |  | 58.60 |  |
| Time in Deceleration Zones: 3 - 4 m/s/s (secs) |  | Control Group |  | 3.86882 |  | 4.4357 |  | 0.00000 |  | 31.10 |  |
|  |  | SSGs+HIIT-Group |  | 3.48652 |  | 4.1069 |  | 0.00000 |  | 29.10 |  |
| Time in Deceleration Zones: > 4 m/s/s (secs) |  | Control Group |  | 1.26835 |  | 2.1189 |  | 0.00000 |  | 17.10 |  |
|  |  | SSGs+HIIT-Group |  | 1.08809 |  | 1.6947 |  | 0.00000 |  | 10.80 |  |
| Distance in Acceleration Zones: 0 - 1 m/s/s (metres) |  | Control Group |  | 519.94037 |  | 416.1745 |  | 0.00000 |  | 2288.09 |  |
|  |  | SSGs+HIIT-Group |  | 597.92199 |  | 424.5340 |  | 0.00000 |  | 2201.29 |  |
| Distance in Acceleration Zones: 1 - 2 m/s/s (metres) |  | Control Group |  | 100.24540 |  | 98.9292 |  | 0.00000 |  | 567.60 |  |
|  |  | SSGs+HIIT-Group |  | 91.30431 |  | 81.4295 |  | 0.00000 |  | 459.22 |  |
| Distance in Acceleration Zones: 2 - 3 m/s/s (metres) |  | Control Group |  | 31.67065 |  | 28.8095 |  | 0.00000 |  | 180.99 |  |
|  |  | SSGs+HIIT-Group |  | 30.94224 |  | 26.0153 |  | 0.00000 |  | 137.18 |  |
| Distance in Acceleration Zones: 3 - 4 m/s/s (metres) |  | Control Group |  | 8.34099 |  | 8.4660 |  | 0.00000 |  | 56.38 |  |
|  |  | SSGs+HIIT-Group |  | 8.87105 |  | 8.2627 |  | 0.00000 |  | 48.41 |  |
| Distance in Acceleration Zones: > 4 m/s/s (metres) |  | Control Group |  | 1.94898 |  | 2.5624 |  | 0.00000 |  | 17.05 |  |
|  |  | SSGs+HIIT-Group |  | 2.23664 |  | 2.5162 |  | 0.00000 |  | 15.26 |  |
| Time in Acceleration Zones: 0 - 1 m/s/s (secs) |  | Control Group |  | 673.13933 |  | 283.4984 |  | 0.00000 |  | 1770.40 |  |
|  |  | SSGs+HIIT-Group |  | 653.75391 |  | 325.1009 |  | 0.00000 |  | 2426.70 |  |
| Time in Acceleration Zones: 1 - 2 m/s/s (secs) |  | Control Group |  | 61.48657 |  | 38.4532 |  | 0.00000 |  | 228.90 |  |
|  |  | SSGs+HIIT-Group |  | 54.65117 |  | 36.5421 |  | 0.00000 |  | 189.00 |  |
| Time in Acceleration Zones: 2 - 3 m/s/s (secs) |  | Control Group |  | 16.92374 |  | 11.7473 |  | 0.00000 |  | 75.40 |  |
|  |  | SSGs+HIIT-Group |  | 16.50078 |  | 11.8008 |  | 0.00000 |  | 58.90 |  |
| Time in Acceleration Zones: 3 - 4 m/s/s (secs) |  | Control Group |  | 4.67866 |  | 4.0879 |  | 0.00000 |  | 25.70 |  |
|  |  | SSGs+HIIT-Group |  | 5.01406 |  | 4.1621 |  | 0.00000 |  | 23.60 |  |
| Time in Acceleration Zones: > 4 m/s/s (secs) |  | Control Group |  | 1.16667 |  | 1.4613 |  | 0.00000 |  | 8.60 |  |
|  |  | SSGs+HIIT-Group |  | 1.35117 |  | 1.4668 |  | 0.00000 |  | 8.60 |  |
| Distance in Power Zone: 0 - 5 SSGs/kg (metres) |  | Control Group |  | 426.11960 |  | 183.6660 |  | 0.00000 |  | 1038.15 |  |
|  |  | SSGs+HIIT-Group |  | 430.25136 |  | 234.5082 |  | 0.00000 |  | 1234.74 |  |
| Distance in Power Zone: 5 - 10 SSGs/kg (metres) |  | Control Group |  | 456.73090 |  | 364.6658 |  | 0.00000 |  | 1937.98 |  |
|  |  | SSGs+HIIT-Group |  | 415.67672 |  | 335.1763 |  | 0.00000 |  | 1605.98 |  |
| Distance in Power Zone: 10 - 15 SSGs/kg (metres) |  | Control Group |  | 241.89529 |  | 265.5062 |  | 0.00000 |  | 1439.56 |  |
|  |  | SSGs+HIIT-Group |  | 304.84141 |  | 303.0984 |  | 0.00000 |  | 1439.57 |  |
| Distance in Power Zone: 15 - 20 SSGs/kg (metres) |  | Control Group |  | 122.25184 |  | 176.6006 |  | 0.00000 |  | 947.19 |  |
|  |  | SSGs+HIIT-Group |  | 186.26953 |  | 246.2531 |  | 0.00000 |  | 1331.71 |  |
| Distance in Power Zone: 20 - 25 SSGs/kg (metres) |  | Control Group |  | 63.63872 |  | 97.9102 |  | 0.00000 |  | 571.43 |  |
|  |  | SSGs+HIIT-Group |  | 102.94326 |  | 176.6684 |  | 0.00000 |  | 1218.03 |  |
| Distance in Power Zone: 25 - 30 SSGs/kg (metres) |  | Control Group |  | 34.49415 |  | 57.2577 |  | 0.00000 |  | 372.20 |  |
|  |  | SSGs+HIIT-Group |  | 48.04812 |  | 80.2448 |  | 0.00000 |  | 522.15 |  |
| Distance in Power Zone: 30 - 35 SSGs/kg (metres) |  | Control Group |  | 19.35418 |  | 31.2944 |  | 0.00000 |  | 201.97 |  |
|  |  | SSGs+HIIT-Group |  | 23.28267 |  | 31.7801 |  | 0.00000 |  | 162.87 |  |
| Distance in Power Zone: 35 - 40 SSGs/kg (metres) |  | Control Group |  | 12.24297 |  | 19.4968 |  | 0.00000 |  | 138.80 |  |
|  |  | SSGs+HIIT-Group |  | 12.73849 |  | 16.0034 |  | 0.00000 |  | 86.03 |  |
| Distance in Power Zone: 40 - 45 SSGs/kg (metres) |  | Control Group |  | 8.46208 |  | 12.9829 |  | 0.00000 |  | 81.78 |  |
|  |  | SSGs+HIIT-Group |  | 8.34210 |  | 10.5497 |  | 0.00000 |  | 63.17 |  |
| Distance in Power Zone: 45 - 50 SSGs/kg (metres) |  | Control Group |  | 6.08858 |  | 10.0353 |  | 0.00000 |  | 73.52 |  |
|  |  | SSGs+HIIT-Group |  | 5.86372 |  | 7.6483 |  | 0.00000 |  | 47.05 |  |
| Distance in Power Zone: > 50 SSGs/kg (metres) |  | Control Group |  | 13.87718 |  | 23.0132 |  | 0.00000 |  | 179.73 |  |
|  |  | SSGs+HIIT-Group |  | 13.32179 |  | 18.4463 |  | 0.00000 |  | 116.14 |  |
| Time in Power Zone: 0 - 5 SSGs/kg (secs) |  | Control Group |  | 990.64101 |  | 403.1155 |  | 0.00000 |  | 2722.10 |  |
|  |  | SSGs+HIIT-Group |  | 942.05859 |  | 480.8834 |  | 0.00000 |  | 2740.10 |  |
| Time in Power Zone: 5 - 10 SSGs/kg (secs) |  | Control Group |  | 275.16331 |  | 214.6909 |  | 0.00000 |  | 1156.20 |  |
|  |  | SSGs+HIIT-Group |  | 249.48672 |  | 198.9130 |  | 0.00000 |  | 998.10 |  |
| Time in Power Zone: 10 - 15 SSGs/kg (secs) |  | Control Group |  | 91.50168 |  | 93.7614 |  | 0.00000 |  | 509.50 |  |
|  |  | SSGs+HIIT-Group |  | 109.11172 |  | 101.5390 |  | 0.00000 |  | 475.10 |  |
| Time in Power Zone: 15 - 20 SSGs/kg (secs) |  | Control Group |  | 36.67842 |  | 49.1308 |  | 0.00000 |  | 271.50 |  |
|  |  | SSGs+HIIT-Group |  | 52.59160 |  | 64.3162 |  | 0.00000 |  | 331.50 |  |
| Time in Power Zone: 20 - 25 SSGs/kg (secs) |  | Control Group |  | 16.95827 |  | 23.4955 |  | 0.00000 |  | 136.50 |  |
|  |  | SSGs+HIIT-Group |  | 24.48281 |  | 35.5717 |  | 0.00000 |  | 234.50 |  |
| Time in Power Zone: 25 - 30 SSGs/kg (secs) |  | Control Group |  | 8.89017 |  | 12.7996 |  | 0.00000 |  | 81.00 |  |
|  |  | SSGs+HIIT-Group |  | 11.20273 |  | 15.2227 |  | 0.00000 |  | 91.60 |  |
| Time in Power Zone: 30 - 35 SSGs/kg (secs) |  | Control Group |  | 5.02878 |  | 6.9516 |  | 0.00000 |  | 44.20 |  |
|  |  | SSGs+HIIT-Group |  | 5.67539 |  | 6.5300 |  | 0.00000 |  | 34.10 |  |
| Time in Power Zone: 35 - 40 SSGs/kg (secs) |  | Control Group |  | 3.18753 |  | 4.3518 |  | 0.00000 |  | 28.00 |  |
|  |  | SSGs+HIIT-Group |  | 3.28066 |  | 3.5901 |  | 0.00000 |  | 19.60 |  |
| Time in Power Zone: 40 - 45 SSGs/kg (secs) |  | Control Group |  | 2.17458 |  | 2.9524 |  | 0.00000 |  | 18.10 |  |
|  |  | SSGs+HIIT-Group |  | 2.17559 |  | 2.4044 |  | 0.00000 |  | 14.00 |  |
| Time in Power Zone: 45 - 50 SSGs/kg (secs) |  | Control Group |  | 1.53597 |  | 2.2115 |  | 0.00000 |  | 15.00 |  |
|  |  | SSGs+HIIT-Group |  | 1.52148 |  | 1.7757 |  | 0.00000 |  | 9.70 |  |
| Time in Power Zone: > 50 SSGs/kg (secs) |  | Control Group |  | 3.61175 |  | 5.3235 |  | 0.00000 |  | 40.60 |  |
|  |  | SSGs+HIIT-Group |  | 3.63223 |  | 4.4451 |  | 0.00000 |  | 25.90 |  |
| Time in HR Load Zone 0% - 60% Max HR(secs) |  | Control Group |  | 506.58945 |  | 495.7649 |  | 0.00000 |  | 2819.80 |  |
|  |  | SSGs+HIIT-Group |  | 408.42695 |  | 475.0961 |  | 0.00000 |  | 2749.10 |  |
| Time in HR Load Zone 60% - 75% Max HR (secs) |  | Control Group |  | 483.46187 |  | 386.9651 |  | 0.00000 |  | 1729.20 |  |
|  |  | SSGs+HIIT-Group |  | 465.49219 |  | 392.7954 |  | 0.00000 |  | 1735.90 |  |
| Time in HR Load Zone 75% - 85% Max HR (secs) |  | Control Group |  | 218.71031 |  | 249.0144 |  | 0.00000 |  | 1115.10 |  |
|  |  | SSGs+HIIT-Group |  | 268.64805 |  | 320.3995 |  | 0.00000 |  | 1593.00 |  |
| Time in HR Load Zone 85% - 96% Max HR (secs) |  | Control Group |  | 173.20288 |  | 373.3207 |  | 0.00000 |  | 2161.90 |  |
|  |  | SSGs+HIIT-Group |  | 231.35137 |  | 391.0727 |  | 0.00000 |  | 2034.20 |  |
| Time in HR Load Zone 96% - 100% Max HR (secs) |  | Control Group |  | 29.61223 |  | 191.5375 |  | 0.00000 |  | 2342.10 |  |
|  |  | SSGs+HIIT-Group |  | 31.24785 |  | 145.4995 |  | 0.00000 |  | 1472.60 |  |
| Accelerations Zone Count: 0 - 1 m/s/s |  | Control Group |  | 0.00000 |  | 0.0000 |  | 0 |  | 0 |  |
|  |  | SSGs+HIIT-Group |  | 0.00000 |  | 0.0000 |  | 0 |  | 0 |  |
| Accelerations Zone Count: 1 - 2 m/s/s |  | Control Group |  | 35.01918 |  | 28.1282 |  | 0 |  | 164 |  |
|  |  | SSGs+HIIT-Group |  | 31.27930 |  | 25.4643 |  | 0 |  | 128 |  |
| Accelerations Zone Count: 2 - 3 m/s/s |  | Control Group |  | 25.10072 |  | 19.1120 |  | 0 |  | 103 |  |
|  |  | SSGs+HIIT-Group |  | 23.27734 |  | 20.3047 |  | 0 |  | 97 |  |
| Accelerations Zone Count: 3 - 4 m/s/s |  | Control Group |  | 10.87770 |  | 9.7128 |  | 0 |  | 68 |  |
|  |  | SSGs+HIIT-Group |  | 11.14453 |  | 9.5736 |  | 0 |  | 50 |  |
| Accelerations Zone Count: > 4 m/s/s |  | Control Group |  | 4.39808 |  | 5.2381 |  | 0 |  | 32 |  |
|  |  | SSGs+HIIT-Group |  | 5.12891 |  | 5.2057 |  | 0 |  | 29 |  |
| Deceleration Zone Count: 0 - 1 m/s/s |  | Control Group |  | 0.00000 |  | 0.0000 |  | 0 |  | 0 |  |
|  |  | SSGs+HIIT-Group |  | 0.00000 |  | 0.0000 |  | 0 |  | 0 |  |
| Deceleration Zone Count: 1 - 2 m/s/s |  | Control Group |  | 34.14149 |  | 27.7723 |  | 0 |  | 153 |  |
|  |  | SSGs+HIIT-Group |  | 32.50781 |  | 26.6092 |  | 0 |  | 140 |  |
| Deceleration Zone Count: 2 - 3 m/s/s |  | Control Group |  | 23.95204 |  | 17.9426 |  | 0 |  | 104 |  |
|  |  | SSGs+HIIT-Group |  | 22.48828 |  | 18.8092 |  | 0 |  | 85 |  |
| Deceleration Zone Count: 3 - 4 m/s/s |  | Control Group |  | 9.61631 |  | 9.3421 |  | 0 |  | 56 |  |
|  |  | SSGs+HIIT-Group |  | 8.94727 |  | 9.5975 |  | 0 |  | 61 |  |
| Deceleration Zone Count: > 4 m/s/s |  | Control Group |  | 4.66187 |  | 6.8295 |  | 0 |  | 50 |  |
|  |  | SSGs+HIIT-Group |  | 4.25000 |  | 5.9456 |  | 0 |  | 37 |  |
|  | | | | | | | | | | | |
